# Statins are associated with reduced likelihood of sarcopenia in a sample of heart failure outpatients: a cross-sectional study

**DOI:** 10.1186/s12872-022-02804-5

**Published:** 2022-08-05

**Authors:** Rui Valdiviesso, Ana Rita Sousa-Santos, Luís F. Azevedo, Emília Moreira, Teresa F. Amaral, José Silva-Cardoso, Nuno Borges

**Affiliations:** 1 FCNAUP - Faculdade de Ciências da Nutrição e Alimentação da Universidade do Porto, Rua do Campo Alegre, 823, 4150-180 Porto, Portugal; 2 CINTESIS - Centro de Investigação em Tecnologias e Serviços de Saúde, R. Dr. Plácido da Costa, s/n, ed. Nascente, piso 2, 4200-450 Porto, Portugal; 3 MEDCIS/FMUP - Departamento de Medicina da Comunidade, Informação e Decisão em Saúde, Faculdade de Medicina da Universidade do Porto, Porto, Portugal; 4 RISE - Rede de Investigação em Saúde, Porto, Portugal; 5 UISPA, LAETA-INEGI/FEUP - Unidade de Investigação de Integração de Sistemas e Unidade de Automação de Processos, Laboratório Associado em Energia, Transportes e Aeronáutica, Faculdade de Engenharia da Universidade do Porto, Instituto de Ciência e Inovação em Engenharia, Porto, Portugal; 6 DM/FMUP - Departamento de Medicina, Faculdade de Medicina da Universidade do Porto, Porto, Portugal; 7 SC/CHUSJ - Serviço de Cardiologia, Centro Hospitalar Universitário de São João, Porto, Portugal

**Keywords:** Heart failure, Sarcopenia, Statins, Endothelial dysfunction, Polypharmacy, Nutritional status

## Abstract

**Background:**

Sarcopenia is prevalent in heart failure (HF) patients, contributing to its poor prognosis. Statin use is postulated as a probable risk for developing sarcopenia, but little is known regarding this association in HF patients. This work aims at classifying and characterising sarcopenia and at describing the association of statin use with sarcopenia in a sample of Portuguese HF outpatients.

**Methods:**

In this cross-sectional study, a sample of 136 HF patients (median age: 59 years, 33.8% women) was recruited from an HF outpatients’ clinic of a University Hospital in Portugal. Sarcopenia was defined according to the European Working Group on Sarcopenia in Older People 2. Clinical, nutritional, and dietary data were collected.

**Results:**

A total of 25 (18.4%) individuals were categorised as sarcopenic, ranging from 12.2% in younger (< 65 years) participants vs. 30.4% in older ones and from 3.3% in men vs. 47.8% in women. Severe sarcopenia accounted for 7.4% of the sample and sarcopenic obesity was identified in 5.1% of the individuals. A total of 65.4% of the participants were statin users. In multivariable analysis (n = 132, 25 sarcopenic), the use of statins was inversely associated with sarcopenia (OR = 0.03; 95% CI = 0.01, 0.30). Each additional age year was associated with a 9% increase in the likelihood of being sarcopenic (OR = 1.09; 95% CI = 1.01, 1.17), and each Kg.m^−2^ increment in body mass index was associated with a 21% decrease in the likelihood of sarcopenia (OR = 0.79; 95% CI = 0.65, 0.96). The daily use of five or more medicines was also directly associated with sarcopenia (OR = 26.87; 95% CI = 2.01, 359.26). On the other hand, being a man and being physically active were inversely associated with sarcopenia (OR = 0.01; 95% CI = 0.00, 0.07 and OR = 0.09; 95% CI = 0.01, 0.65, respectively).

**Conclusions:**

Contrary to what was expected, patients medicated with statins were less likely to be sarcopenic. Although this finding deserves further research, we hypothesise that this might be related to the pleiotropic effects of statins on endothelial function, contributing to better neuromuscular fitness.

## Introduction

Sarcopenia can be defined as a progressive skeletal muscle disease that increases the likelihood of adverse outcomes such as disability, falls, fractures and mortality. It is characterised by the loss of muscle strength and muscle quantity or quality. Low physical performance further classifies sarcopenia as severe [1].

Heart failure (HF) and sarcopenia share pathophysiological pathways involving muscle dysfunction that include alterations in mitochondrial density and activity, fibre distribution and oxidative stress. Both diseases contribute to physical inactivity, which in turn aggravates both cardiac and muscular status [2]. Sarcopenia is considered one of the leading causes of reduced cardiorespiratory fitness and poor physical performance in HF patients [3], and contributes to mortality in older HF patients [4]. The pooled prevalence of sarcopenia in HF patients ranges from 55% in hospitalised patients to 26% in community-dwelling ones, with an overall pooled prevalence of 34% [5].

Statin use is often designated as a probable risk for developing sarcopenia [6]. Statins are competitive inhibitors to the 3-Hydroxy-3-methylglutaryl-coenzyme A reductase, a hepatic converter of an early phase cholesterol precursor, thus contributing to lower serum cholesterol levels [7]. Until recently, the prescription of statins to HF patients remained controversial, as studies evaluating cardiovascular outcomes show conflicting results [8]. However, a recent meta-analysis by Bielecka-Dabrowa et al. including 17 clinical trials and cohort studies showed a reduction in cardiovascular mortality, all-cause mortality and hospitalisation in patients undergoing statin therapy when compared with non-statin users. This effect was independent of HF aetiology and ejection fraction. The same study concluded that lipophilic statins seem to be more favourable than hydrophilic ones [9].

The 2021 guidelines of the European Society of Cardiology for the treatment of HF do not recommend initiating statin therapy in patients with HF with reduced ejection fraction and only support the continuation of statin use in HF patients with coronary artery disease and/or hyperlipidaemia [10], a reason why most clinicians do not suspend statins in patients with these conditions that further develop HF [11].

The potential prosarcopenic properties of statins are related to statin-mediated mechanisms of muscle dysfunction involving inflammation, apoptosis, the ubiquitin–proteasome system, insulin-like growth factor 1 and myostatin [8]. Muscle complications such as myalgia, myopathy and rhabdomyolysis are the most described side-effects of statin use [12] and are the main reasons for suspending statin therapy [13]. It has also been postulated that the potential prosarcopenic effects of statins could limit their effectiveness in HF patients [8].

Despite the described potential risks, the effect of statins on the muscular mass and function in HF patients, and namely in their sarcopenia status, remains controversial. With this study, we aim at classifying and characterising sarcopenia and at describing the association of statin use with sarcopenia in a sample of Portuguese HF outpatients.

## Methods

**Fig. 1:**
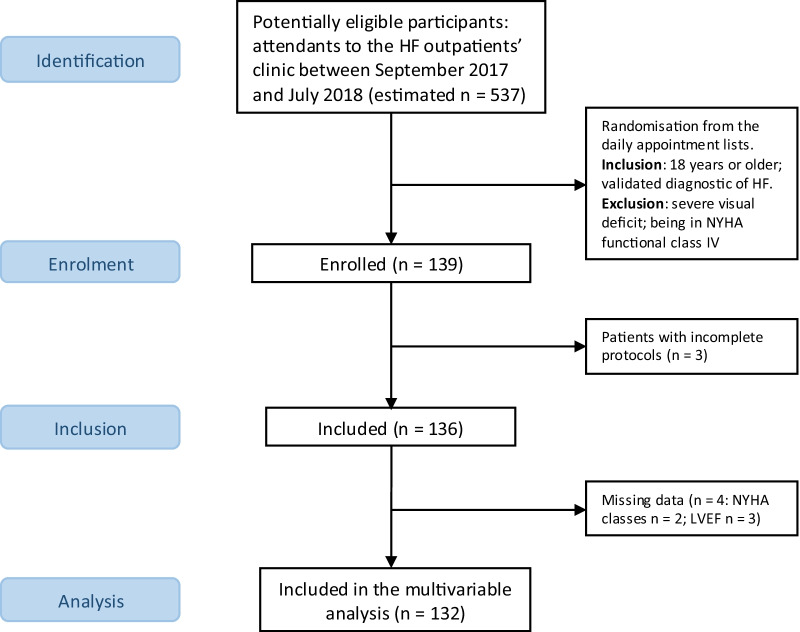
Flow diagram of the study

Participants in this cross-sectional study were randomly recruited from the appointment lists of an outpatients HF and transplantation clinic of a Portuguese university hospital. Due to a lack of information regarding the number of attendees to the HF appointments for the period the data was collected (September 2017 to July 2018), we estimated the number of potentially eligible patients at 537, based on a study conducted in the same setting in a similar period [14]. Inclusion and exclusion criteria were applied at recruitment. Patients were included if they were 18 years or older and had a clinically validated diagnostic of HF [10]. Patients with severe visual impairment were excluded, as well as patients within the New York Heart Association (NYHA) functional class IV, due to their limitations in complying with the study protocol. The flow diagram is shown in Fig. 1.

All anthropometric measurements were performed by a registered nutritionist according to standard procedures and are thoroughly described elsewhere [15]. These measurements include standing height, weight, calf circumference, mid-upper arm circumference (MUAC) and triceps skinfold thickness (TST). Mid-upper arm muscle circumference (MAMC) was calculated using the Jelliffe Eq. [16]: MAMC = MUAC-(3.14 × TST). Body mass index (BMI), in Kg.m^−2^, was calculated using the standard formula: Weight (Kg)/standing height (m)^2^.

Sarcopenia was defined according to the European Working Group on Sarcopenia in Older People 2 (EWGSOP2) guidelines and algorithm for diagnosis [1]. The average of three dynamometer compressions at the non-dominant hand was used to assess muscle strength, using a calibrated electronic hand dynamometer (Jamar Plus +) and following the measurement procedures of the American Society of Hand Therapists [17]. Cut points for classifying low hand grip strength were defined as < 27 Kgf for men and < 16 Kgf for women [18]. Low muscle quantity was defined as calf circumference < 31 cm [19], or MAMC < 21.1 cm for men and < 19.2 cm for women [20]. Usual gait speed ≤ 0.8 m.s^−1^ was used to classify the severity of sarcopenia [1]. Sarcopenic obesity was defined as the coexistence of sarcopenia with BMI ≥ 30 kg.m^−2^ [21].

HF clinical status was assessed by cardiologists. Medical records were also consulted. Data included left-ventricular ejection fraction (LVEF) percentage and phenotypes of heart failure defined as heart failure with reduced ejection fraction (HFrEF), heart failure with mildly reduced ejection fraction (HFmrEF) and heart failure with preserved ejection fraction (HFpEF) [10], functional HF classes according to the NYHA [22], HF aetiology, atrial fibrillation, incident myocardial infarction, type 2 diabetes mellitus, and medication. Polypharmacy was classified as the daily concurrent use of five or more different medicines [23].

Physical activity was evaluated using the International Physical Activity Questionnaire – Short Form, validated for the Portuguese population [24], and categories were defined as inactive, minimally active and active [25].

Daily energy and macronutrient intake were estimated using a 24-h dietary recall [26] conducted by trained nutritionists, with visual aids for tableware and food portions [27]. Missing portion information was complemented with usual portions or product brand names using normative tables of food weights and portions [28]. The recall registers were converted into nutrients using the Portuguese Food Composition Table [29]. For the multivariable analysis, total daily fat intake was adjusted for total body weight (g/Kg/day).

### Statistical analysis

The sample was described according to the sarcopenia status and the use of statins. Quantitative variables were tested for distribution using Shapiro–Wilk test and associated with the outcome categories using parametric and non-parametric tests. Categorical data were compared using chi-square or Fisher’s exact tests as adequate. Bonferroni adjustment was used to assess significant differences in categorical subsets. Results are presented as number and percentage [n (%)] for categorical variables, mean and standard deviation [M (SD)] for normally-distributed variables and median and inter-quartile range [Md (IQR)] for variables with skewed distribution.

A logistic regression was carried out for a total of 132 participants, as four patients with missing values (HF LVEF classification, n = 3; NYHA functional classification, n = 2), were withdrawn from the multivariable analysis. There was no difference in the predictive ability for all independent variables between the presented model and the complete analysis (with 136 participants and ignoring the missing values), and differences between β-coefficients of the two models were very low, ranging from -0.05 to 0.04, with an average difference of 0.01.

The following predictors for having sarcopenia were included in the multivariable model: statin use as the main predictor; age and sex as usual confounders; HF phenotypes according to the LVEF (HFpEF, HFmrEF, HFrEF) and NYHA functional classes I, II and III, as important indicators of HF clinical status; HF aetiology, coded as “ischaemic vs. others”, to rule out selection bias as to the indication for statin treatment; total daily fat intake per Kg as a surrogate of dietary therapeutic recommendations towards reduction in fat intake that usually accompany statin treatment; polypharmacy, which is usually associated with sarcopenia; physical activity, which may be reduced in sarcopenic patients; and BMI as a measure of weight status and a surrogate of possible wasting. Linearity between continuous variables was assessed using Box-Tidwell test. Model performance was evaluated using Omnibus likelihood ratio chi-square test, Hosmer and Lemeshow test and Nagelkerke R-square. Odds ratios (OR) and respective 95% confidence intervals (95% CI) were calculated. All tests were performed for a level of statistical significance of *p* < 0.050, and using IBM SPSS Statistics version 27.

A post hoc power calculation was carried out regarding the measure of association between statin use and sarcopenia [30, 31]. The initial power (1-β) to detect the association between statin use and sarcopenia (OR = 0.39; 95% CI = 0.16, 0.96; *p* = 0.040) was defined at 63%, given the sample size of n = 132, a type-1 error rate of 5%, the proportion of sarcopenic patients exposed to statins of 0.48, the proportion of robust patients exposed to statins of 0.70 and the proportion of sarcopenic/robust patients of 0.23.

## Results

Overall, 136 patients (median age 59 years, 33.8% women) were included in this study. The sample is characterized in Table 1: a total of 25 (18.4%) individuals were categorised as sarcopenic, ranging from 12.2% in younger (< 65 years) participants vs. 30.4% in older ones (*p* = 0.009) and from 3.3% in men vs. 47.8% in women (*p* < 0.001). Severe sarcopenia accounted for 7.4% of the sample and sarcopenic obesity was present in 5.1% of the participants.

**Table 1:** Characteristics of the sample according to the presence of sarcopenia

	Normal (n = 111)	Sarcopenic (n = 25)	*p*-value
Sex, n (%)			< 0.001
Women	24 (21.6)	22 (88.0)	
Men	87 (78.4)	3 (12.0)	
Age, Md (IQR)	58.0 (49.0, 67.0)	67.0 (52.0, 70.5)	0.038
Age intervals, n (%)			0.009
< 65	79 (71.2)	11 (44.0)	
≥ 65	32 (28.8)	14 (56.0)	
HF aetiology, n (%)			0.040
Dilated cardiomyopathy	59 (54.6)	13 (56.5)	
Ischaemic	33 (30.6)	5 (21.7)	
Myocarditis	5 (4.6)	0 (0.0)	
Hypertrophic cardiomyopathy*	4 (3.7)	5 (21.7)	
Others	7 (6.5)	0 (0.0)	
LVEF, %, M (SD)	36.8 (12.9)	42.3 (16.5)	0.080
LVEF categories, n (%)			0.202
HFrEF (< 40%)	56 (51.9)	10 (40.0)	
HFmrEF (40–50%)	31 (28.7)	6 (24.0)	
HFpEF (≥ 50%)	21 (19.4)	9 (36.0)	
NYHA classification, n (%)			0.189
Class I	42 (38.5)	5 (20.0)	
Class II	49 (45.0)	16 (64.0)	
Class III	18 (16.5)	4 (16.0)	
Medications, n (%)			
ACE inhibitors	87 (79.1)	19 (76.0)	0.734
Beta blockers	106 (96.4)	23 (92.0)	0.339
Aldosterone antagonists	77 (70.0)	14 (56.0)	0.178
Statins	77 (69.4)	12 (48.0)	0.042
Furosemide	37 (33.6)	14 (56.0)	0.037
Incident myocardial infarction, n (%)	28 (25.7)	4 (16.7)	0.436
Atrial fibrillation, n (%)	16 (15.1)	3 (12.0)	0.999
Type 2 Diabetes Mellitus	33 (30.0)	6 (24.0)	0.550
Polypharmacy, n (%)	79 (71.8)	22 (88.0)	0.092
Physical activity, n (%)			0.017
Inactive*	56 (50.5)	20 (80.0)	
Minimally active	41 (36.9)	5 (20.0)	
Active	14 (12.6)	0 (0.0)	
Weight, Kg, Md (IQR)	80.8 (73.5, 89.6)	67.2 (56.7, 73.9)	< 0.001
Standing height, cm, M (SD)	166.9 (8.6)	153.8 (8.8)	< 0.001
BMI, Kg.m^−2^, M (SD)	29.5 (4.2)	28.0 (4.7)	0.120
BMI classes, n (%)			0.118
Underweight + Normal	17 (15.3)	8 (32.0)	
Overweight	46 (41.4)	10 (40.0)	
Obese	48 (43.2)	7 (28.0)	
Hand grip strength, Kgf, Md (IQR)	32.7 (26.5, 39.5)	18.0 (16.2, 22.9)	< 0.001
Gait speed, m.s^−1^, Md (IQR)	1.13 (0.93, 1.31)	0.83 (0.73, 1.07)	< 0.001
Gait speed ≤ 0.8 m.s^−1^, n (%)	5 (4.5)	10 (40.0)	< 0.001
Dietary assessment			
Energy, Kcal/day, Md (IQR)	1765 (1500, 2227)	1533 (1151, 1792)	0.008
Total fat, g/day, Md (IQR)	56.9 (42.5, 86.2)	48.2 (31.2, 61.6)	0.020
Carbohydrates, g/day, Md (IQR)	198.1 (158.5, 245.8)	183.5 (118.8, 212.3)	0.069
Protein, g/day, Md (IQR)	85.9 (60.4, 106.3)	70.9 (59.1, 99.5)	0.134

Values are presented as: n (%) = number (percentage); M (SD) = Mean (Standard Deviation); Md (IQR) = Median (Lower quartile, Upper quartile). HF = Heart Failure; LVEF = Left Ventricular Ejection Fraction; HFrEF = Heart Failure with reduced Ejection Fraction; HFmrEF = Heart Failure with mildly reduced Ejection Fraction; HFpEF = Heart Failure with preserved Ejection Fraction; NYHA = New York Heart Association functional HF classes. ACE = Angiotensin-conversion Enzyme; BMI = Body Mass Index. Missing values: LVEF n = 3; NYHA n = 2; Incident myocardial infarction n = 2; Atrial fibrillation n = 5

*Results differ significantly between subsets of dependent variable, as per Bonferroni adjusted *p*-values

Participants with an aetiological diagnosis of hypertrophic cardiomyopathy were more likely to be sarcopenic (*p* = 0.040), as well as physically inactive (*p* = 0.017) individuals. Estimated total energy and fat intakes were also lower in sarcopenic participants (*p* = 0.008 and *p* = 0.020 respectively).

The use of statins in this sample is described in Table 2, including the variables entered in the multivariable model. A total of 65.4% of the participants were statin users. As expected, ischaemia and stroke were related to statin medication (*p* < 0.001). The proportion of older HF patients medicated with statins was higher than those that were not statin users (40.4% vs. 21.3%, *p* = 0.025). Statin users are also those who used more daily medication (*p* < 0.001).

**Table 2:** Characteristics of the sample according to the use of statins

	Not medicated with statins (n = 47)	Statin users (n = 89)	*p*-value
Sex, n (%)			0.237
Women	19 (40.4)	27 (30.3)	
Men	28 (56.9)	62 (67.7)	
Age, years, Md (IQR)	51.0 (39.0, 64.0)	62.0 (54.0, 69.0)	< 0.001
Age categories, n (%)			0.025
< 65 years	37 (78.7)	53 (59.6)	
≥ 65 years	10 (21.3)	36 (40.4)	
HF aetiology, n (%)			< 0.001
Dilated cardiomyopathy	29 (61.7)	43 (48.3)	
Ischaemic*	4 (8.5)	34 (38.2)	
Myocarditis*	4 (8.5)	1 (1.1)	
Hypertrophic cardiomyopathy	4 (8.5)	5 (5.6)	
Others	6 (12.8)	6 (6.7)	
LVEF categories, n (%)			0.404
HFrEF + HFmrEF	36 (78.3)	63 (71.6)	
HFpEF	10 (21.7)	25 (28.4)	
NYHA classification, n (%)			0.455
Class I	16 (34.8)	31 (35.2)	
Class II	20 (43.5)	45 (51.1)	
Class III	10 (21.7)	12 (13.6)	
Incident myocardial infarction, n (%)	2 (4.5)	30 (33.7)	< 0.001
Polypharmacy, n (%)	25 (54.3)	76 (85.4)	< 0.001
Type 2 Diabetes Mellitus	9 (19.6)	30 (33.7)	0.086
Number of medicines/day, Md (IQR)	5.0 (3.8, 8.0)	7.0 (5.5, 9.5)	< 0.001
Physical activity, n (%)			0.175
Inactive	30 (63.8)	46 (51.7)	
Minimally active + active	17 (36.2)	43 (48.3)	
Body Mass Index, Kg.m^−2^, M (SD)	28.9 (4.7)	29.4 (4.2)	0.526
Fat intake, g/day, Md (IQR)	59.7 (47.5, 86.4)	54.1 (37.0, 74.4)	0.149

Values are presented as: n (%) = number (percentage); M (SD) = Mean (Standard Deviation); Md (IQR) = Median (Lower quartile, Upper quartile). HF = Heart Failure; LVEF = Left Ventricular Ejection Fraction; HFrEF = Heart Failure with reduced Ejection Fraction; HFmrEF = Heart Failure with mildly reduced Ejection Fraction; HFpEF = Heart Failure with preserved Ejection Fraction. Missing values: LVEF n = 3; NYHA n = 2; Incident myocardial infarction n = 2

*Results differ significantly between subsets of dependent variable, as per Bonferroni adjusted *p*-values

The multivariable model (n = 132, 25 sarcopenic) was able to correctly classify 91.7% of the cases and to explain 70.6% of the variance (Nagelkerke R-square) in sarcopenia. Table 3 depicts the results of the logistic regression. The use of statins was inversely associated with sarcopenia (OR = 0.03; 95% CI = 0.01, 0.30). Each additional age year was associated with a 9% increase in the likelihood of being sarcopenic (OR = 1.09; 95% CI = 1.01, 1.17), and each Kg.m^−2^ increment in BMI was associated with a 21% decrease in the likelihood of sarcopenia (OR = 0.79; 95% CI = 0.65, 0.96). The daily use of five or more medicines was also directly associated with sarcopenia (OR = 26.87; 95% CI = 2.01, 359.26). On the other hand, being a man and being physically active were inversely associated with sarcopenia (OR = 0.01; 95% CI = 0.00, 0.07 and OR = 0.09; 95% CI = 0.01, 0.65, respectively).

**Table 3:** Bivariable and multivariable results from the logistic regression analysis regarding sarcopenia status (n = 132)

	Unadjusted		Adjusted	
	OR (95% CI)	*p*-value	OR (95% CI)	*p*-value
Statin				
No	1		1	
Yes	0.39 (0.16, 0.96)	0.040	0.03 (0.01, 0.30)	0.003
Age, years	1.04 (0.99, 1.08)	0.077	1.09 (1.01, 1.17)	0.022
Sex				
Women	1		1	
Men	0.04 (0.01, 0.14)	< 0.001	0.01 (0.00, 0.07)	< 0.001
LVEF categories				
HFpEF	1		1	
HFmrEF	2.14 (0.66, 6.93)	0.203	5.07 (0.61, 42.14)	0.133
HFrEF	2.40 (0.86, 6.73)	0.096	3.78 (0.60, 25.07)	0.169
NYHA functional classes				
NYHA Class I	1		1	
NYHA Class II	2.74 (0.92, 8.12)	0.068	4.82 (0.41, 56.56)	0.211
NYHA Class III	2.10 (0.50, 8.82)	0.311	14.65 (0.73, 293.72)	0.079
HF aetiology				
Ischaemic	1		1	
Others	1.63 (0.56, 4.73)	0.368	1.30 (0.20, 8.40)	0.781
Polypharmacy				
< 5 medicines/day	1		1	
≥ 5 medicines/day	2.99 (0.85, 10.72)	0.093	26.87 (2.01, 359.26)	0.013
Physical activity				
Inactive	1		1	
Minimally active + active	0.25 (0.09, 0.70)	0.009	0.09 (0.01, 0.65)	0.017
Body mass index, Kg.m^−2^	0.92 (0.82, 1.02)	0.124	0.79 (0.65, 0.96)	0.017
Total fat intake, g/Kg/day	0.72 (0.26, 2.04)	0.538	0.34 (0.02, 5.20)	0.437

Values are expressed in Odds Ratio (OR) and 95% Confidence Intervals (95% CI). HF = Heart Failure; LVEF = Left Ventricular Ejection Fraction; HFrEF = Heart Failure with reduced Ejection Fraction; HFmrEF = Heart Failure with mildly reduced Ejection Fraction; HFpEF = Heart Failure with preserved Ejection Fraction. Omnibus test: *p* < 0.001; Nagelkerke R-square = 0.706; Hosmer and Lemeshow test: *p* = 0.801. Model sensitivity: 76.0%; Model specificity: 95.3%. Model accuracy: 91.7%

NYHA classes, LVEF categories, ischaemic aetiology and daily fat intake were not associated with sarcopenia.

## Discussion

The frequency of sarcopenia in this sample is in line with the global prevalence for HF ambulatory patients [5] when considering the mean age of our population. As no studies are known regarding sarcopenia in HF populations from Portugal, we can only contrast our results with healthy populations, namely with older adults: Sousa-Santos et al., reported a frequency of sarcopenia of 4.4% in a population-based cross-sectional study (Nutrition UP 65, n = 1500) [32], which is significantly lower than our sample’s frequency.

It is worth noticing that 44% of all sarcopenic patients in our sample were younger than 65 years, which highlights the fact that sarcopenia secondary to HF cannot be considered a geriatric syndrome. This reality demands more thorough attention on younger HF patients regarding their muscular mass and function.

### Statins

Contrary to what was expected, in this sample statins were consistently associated with not being sarcopenic. A possible explanation for this probable protective factor might lie in the fact that the benefits of statins at a circulatory level could positively impact the neuromuscular function, thus contributing to the preservation of muscle strength, quantity, and quality. This cardiovascular pleiotropic effect of statins results in an anti-atherogenic state that goes beyond the effects on plasmatic lipids, inhibiting the proliferation of cytokines, c-reactive protein and cellular adhesion molecules and decreasing the adhesion of monocytes to the endothelium [33]. Contributing to endothelial health, statins also indirectly promote nitric oxide release and bioavailability [34].

The association of statin use with better outcomes in HF patients [9] may be related to the known beneficial effects of statins on vascular health [33], as endothelial dysfunction is tightly connected with HF in a bidirectional way: HF promotes endothelial dysfunction and, in turn, the latter promotes the development of the former [35]. This is also the case between HF and sarcopenia [3]. As HF patients with sarcopenia have impaired endothelial function, with lower vasodilation impacting exercise capacity [36], the use of statins could, potentially, improve their endothelial function, which may lead to better muscle perfusion, thus contributing to better neuromuscular fitness.

In fact, the link between endothelial dysfunction and sarcopenia has been gaining traction in the light of recent evidence. A study with 236 rural elderly women showed a significant correlation between endothelial dysfunction and low hand grip strength [37]. A systematic review of publications (n = 18) reported that endothelial dysfunction may be an early predictor of frailty and sarcopenia [38]. In a cohort of chronic kidney disease patients (n = 77), sarcopenia was also more frequent in those with markers of atherosclerosis and endothelial dysfunction [39].

Alcalde-Estévez et al. suggested a probable mechanism for the association of endothelial dysfunction and sarcopenia, using cultured murine myoblasts incubated with endothelin-1, a peptide with augmented expression in endothelial dysfunction, resulting in myoblast senescence and fibrosis [40]. It is known that statins reduce significantly the concentrations of endothelin-1, as demonstrated by a meta-analysis of 15 randomized controlled trials by Sahebkar et al. [41]. These findings contribute to the biological plausibility of our hypothesis that statins may have a pleiotropic protective effect on sarcopenia.

### Physical activity, weight status and fat intake

As expected, active participants had lower odds of being sarcopenic. Exercise is an important factor for primary and secondary prevention of HF and for a better prognosis of the disease [10]. Aerobic and resistance exercise is associated with better quality of life and reduced hospitalisation in HF patients [42]. Similarly, combination exercise has the best prevention and therapeutic effects on sarcopenia [43]. Our results show that physical inactivity is highly frequent in this HF sample, being significantly higher in sarcopenic individuals, which calls for action regarding the recommendation of adequate and tailored exercise training.

The HF-ACTION controlled trial is the only known work studying the influence of statins on exercise training response in HF patients (n = 2331, with LVEF ≤ 35%). This study found no interaction between statin use and changes in exercise capacity or quality of life [44].

Regarding weight status, after adjusting for covariates, BMI was associated with lower odds of being sarcopenic. We attribute this association to the loss of lean body mass typical of sarcopenia [1].

Together with physical activity, changes in dietary habits are usually recommended for patients initiating statin use. Regardless of these recommendations, total fat intake has significantly increased over time in US statin users but not in non-users [45] and diet and lifestyle of Australian statin users were not significantly different from those of non-users, albeit statin users had lower saturated fat intake [46]. In this sample’s bivariable analysis regarding total fat intake, no differences were found between statin vs. non-statin users but sarcopenic patients had significantly lower fat intake. In multivariable analysis, fat intake was not able to predict sarcopenia.

### Polypharmacy

HF treatment demands prescribing multiple medications, in accordance with international guidelines [10], leading to a very high prevalence of polypharmacy. The management of multiple comorbidities associated with HF further increases the number of medicines needed, particularly in older patients, which makes polypharmacy a universal condition in HF populations [47]. While medication that contributes to maintaining haemodynamic stability in HF patients is indispensable, patients could potentially benefit from reducing the use of other medicines that were not directly related to the maintenance or improvement of cardiovascular function or the management of comorbidities. Such is the case of non-prescription drugs, which seem to be of extremely common use by HF patients [48].

Evolution in pharmacological interventions in HF has contributed to reducing mortality and/or hospitalisations in the recent past [49, 50], but may also impose some challenges regarding many clinical aspects and, namely, in sarcopenia: in general, polypharmacy is associated with lower physical function in older adults [51]; polypharmacy was independently associated with sarcopenia in a cross-sectional study with German community-dwelling older adults [52] and with sarcopenia, disability and mortality in a cohort of community-dwelling Japanese older adults [53]. In the present study, polypharmacy may be a surrogate for the coexistence of HF with other comorbidities that are also associated with sarcopenia, such as diabetes, cancer, respiratory disease, kidney disease or cognitive impairment [35, 54]. Apart from diabetes, which was not related to sarcopenic status, we did not include other comorbidities in our study protocol, therefore we were not able to assert this possibility.

In our sample, the daily use of 5 or more medicines was associated with being sarcopenic, whereas the use of statins had the opposite association. Much more evidence is needed regarding the role of statins in HF and either if they should be withheld or withdrawn in the context of reducing polypharmacy or having an effect on neuromuscular fitness.

### Limitations and strengths

This exploratory study has some limitations that the authors would like to acknowledge, starting with the cross-sectional design, which does not allow for causal associations. The small sample size can be considered a major limitation of this research, as it can be a source of increased variability and thus introduce various biases that could hamper the interpretation of the results and undermine the validity of the findings. Despite this, we were able to find a significant association between the use of statins and sarcopenia.

Regarding the classification of sarcopenia, the EWGSOP2 does not recommend the use of anthropometry to assess muscular quantity and quality criteria for diagnosing this condition, except when other methods are not available, such as dual energy X-ray absorptiometry (DEXA) or bioimpedance analysis. In this case, the recommended anthropometric method is the calf circumference [1], which we used to estimate muscle quantity. We decided to additionally use MAMC, as this estimator is based on upper-arm anthropometry, an area that is usually free from oedema, with clear benefits when evaluating HF patients [55, 56], and with the added advantages of having defined and validated sex-specific cut-offs [20] and of being a fast, simple and inexpensive method. A recent study on Portuguese older adults found that calf circumference was shown to have a very high specificity in classifying sarcopenia when compared with reference criteria of muscle mass measurements using DEXA, namely a specificity of 100% contrasted with DEXA-measured appendicular skeletal muscle and of 94% contrasted with DEXA-measured appendicular skeletal muscle adjusted for height. MAMC was not far behind calf circumference, with respective values of sensitivity of 96% and 85%. This shows that anthropometry can be a valid indicator to rule in the presence of sarcopenia when no other method is present [57]. Moreover, all anthropometric measurements were performed by the same trained nutritionist, in order to avoid inter-observer error.

Most of the data regarding the clinical status of the participants were collected from medical records whose purpose was not epidemiological research. Therefore, we were not able to retrieve data on sufficient patients regarding comorbidities such as kidney disease or respiratory disease, and parameters such as LDL cholesterol or N-terminal pro-B-type natriuretic peptide levels.

This study uses dietary intake data produced by a single 24 dietary recall, which does not account for day-to-day variations and can underestimate energetic intake [58]. However, all interviews were made by trained nutritionists who placed special care on the registry of the dietary recalls and used ancillary tools to estimate food portions and undertake the complex conversion of food records into nutritional information.

The type of statins was not registered and the prescribed dose was not analysed. Our sample size would not allow stratification of statin users for type and dose, but it is still worth mentioning that, as lipophilic statins seem to be associated with better HF outcomes when compared with hydrophilic ones and low doses seem to be more beneficial to HF patients [8], dose and lipophilicity of statins should be important factors on future study designs.

Lastly, some bivariable analysis in this sample might suggest a possible survival bias underlying the relations between particular clinical conditions, sex, statin use and sarcopenia. As previously mentioned, the prescription of statins is significantly higher in ischaemic patients, who account for 60.5% of individuals below 65 years vs. 39.5% in older ones. Also, the proportion of ischaemic men is much higher than that of women’s (73.7% vs. 26.3%). As 88.0% of all participants classified with sarcopenia were women, and considering that coronary heart disease and stroke are the main causes of cardiovascular death in HF patients and, mainly, in male HF patients [59], this might mean that women were more likely to survive and to develop sarcopenia.

Despite the aforementioned limitations, this study is, to our knowledge, the first to describe sarcopenia in HF Portuguese patients in regard to their clinical and nutritional status, and the first to quantify the association between the use of statins and a reduced likelihood of sarcopenia in patients with HF. We are not, however, in a position to recommend or advocate for a change of medication towards HF patients. More evidence is needed, especially prospective studies and specifically randomized clinical trials, in order to clarify the complex relations of pharmacological intervention in HF patients, namely those affected by comorbidities associated with nutritional status and, specifically, skeletal muscle, such as sarcopenia and frailty.

Finally, we would like to stress that screening for sarcopenia is not a usually recommended clinical practice, despite its association with worst prognosis both in ageing and in many chronic diseases. Sarcopenia is preventable, manageable, treatable and, in some cases, reversible, and assessing sarcopenia in clinical settings is easy and inexpensive. The EWGSOP2 provides a simple algorithm for case-finding, diagnosis and severity determination [1]. We claim that clinical suspicion of sarcopenia exists in all patients with HF, hence all should be screened, including the younger, and a low hand grip strength value (< 27 Kgf for men and < 16 Kgf for women) should be enough to classify sarcopenia as probable and to start intervention.

## Conclusions

In summary, all HF patients, including younger patients, should be screened and monitored for the onset and evolution of sarcopenia. In this HF sample, statin users were less likely to be sarcopenic than non-users, irrespective of age, sex, LVEF, NYHA classes, ischaemia as HF aetiology, dietary fat intake, polypharmacy, physical activity and weight status. Although this finding deserves further research, we hypothesise that this association might possibly be attributable to the pleiotropic effects of statins on endothelial function, contributing to a better neuromuscular fitness.

## Data Availability

The datasets generated and/or analysed during the current study are not publicly available due to ongoing research based on the same datasets, but are available from the corresponding author on reasonable request.

## Abbreviations

HF: Heart failure
NYHA: New York Heart Association
MUAC: Mid-upper arm circumference
TST: Triceps skinfold thickness
MAMC: Mid-upper arm muscle circumference
BMI: Body mass index
EWGSOP2: European Working Group on Sarcopenia in Older People 2
LVEF: Left-ventricular ejection fraction
HFrEF: Heart failure with reduced ejection fraction
HFmrEF: Heart failure with mildly reduced ejection fraction
HFpEF: Heart failure with preserved ejection fraction
DEXA: Dual energy X-ray absorptiometry
